# Efficacy of Cyclophosphamide treatment for immunoglobulin G4-related disease with addition of glucocorticoids

**DOI:** 10.1038/s41598-017-06520-5

**Published:** 2017-07-21

**Authors:** Fei Yunyun, Chen Yu, Zhang Panpan, Chen Hua, Wu Di, Zhao Lidan, Peng Linyi, Wang Li, Wu Qingjun, Zhang Xuan, Zhao Yan, Zeng Xiaofeng, Zhang Fengchun, Zhang Wen

**Affiliations:** Departments of Rheumatology, Peking Union Medical College Hospital, Clinical Immunology Center, Chinese Academy of Medical Science & Peking Union Medical College, Beijing, China

## Abstract

Aim to evaluate the efficacy and safety of glucocorticoid monotherapy vs combination therapy of cyclophosphamide (CYC) for IgG4 related disease (IgG4-RD). 102 newly diagnosed IgG4-RD patients were enrolled and assigned to 2 groups: Group I was prednisone monotherapy (0.5–1.0 mg/kg.d, tapered gradually) and Group II was glucocorticoid and CYC (50–100 mg per day). Patients were assessed at different periods. Primary end point was relapse rate; secondary end points included response, remission rate and adverse effects. 52 patients were in Group I and 50 in Group II. At 1 month, both groups achieved obvious improvement. Accumulated relapse rate during 1 year was 38.5% in Group 1, including 12 cases with clinical relapse and 8 patients manifesting only serological relapse; whereas there was 12.0% of relapse in Group 2, only 1 with clinical relapse and other 5 patients got serological relapse. The mean flare time in Group II was significantly longer than that in Group I. All relapsing patients in Group I were sensitive to immunosuppressants. Most patients involving more than 6 organs in Group I relapsed during 1 year. IgG4 levels of relapse cases were significantly higher than non-relapsing patients at baseline. Bile duct, lacrimal glands and lymph nodes were commonly relapsed organs in Group I.

## Introduction

IgG4-related disease (IgG4-RD) is a newly recognized systemic disease characterized by multiple sites swelling, which are caused by lymphoplasmacytic infiltration and sclerosis, combined with elevated serum IgG4 levels and infiltration of IgG4 positive plasma cells in the involved organs and tissues^1, 2^. IgG4-RD can be localized to one or two organs or be present with diffuse systemic disease. Previous studies have documented various organs involvement of IgG4-RD, manifesting as the autoimmune pacreatitis, sclerosing cholangitis, sclerosing sialadenitis, sclerosing dacryoadenitis, hepatopathy, retroperitoneal fibrosis, inflammatory aneurysm, IgG4-related lung disease, as well as the inflammatory pseudotumour, etc^1–5^.

Corticosteroid are the standard first-line treatment for IgG4-RD and it is widely accepted that patients with IgG4-RD are generally sensitive to glucocorticoid^6–8^. Unfortunately, despite IgG4-RD responding swiftly to corticosteroid, disease-modifying anti-rheumatic drugs are often required to keep remission because relapse occurred in about 50% patients with corticosteroid tapering or withdrawal^6, 8^. Up to now, it is still uncertain whether immunosuppressants and corticosteroid combination are more efficacious than single corticosteroid therapy for induction therapy and preventing disease flares^8–11^.

As the tertiary referral center in China, Peking Union Medical College Hospital (PUMCH) has recruited newly diagnosed IgG4-RD patients from January 2011 nationwide^12, 13^. In this study, we compared the difference of therapeutic effect, relapse rate, as well as side effect between glucocorticoids monotherapy and combination treatment with glucocorticoids and cyclophosphamide. The results indicated that the addition of cyclophosphamide to glucocorticoids is highly effective, and preventing relapse.

## Methods

All methods were carried out in accordance with relevant guidelines and regulations. The study protocol was approved by the Ethics Committee of Peking Union Medical College Hospital. All enrolled patients consented to attend this cohort study and signed written informed consent.

### Enrollment

In our prospective cohort study of IgG4-RD performed by the Peking Union Medical College Hospital^12, 13^, 248 patients were enrolled between January 2011 and December 2014, which was registered as ClinicalTrials.gov ID: NCT01670695 at August 20th, 2012.

**Figure 1:**
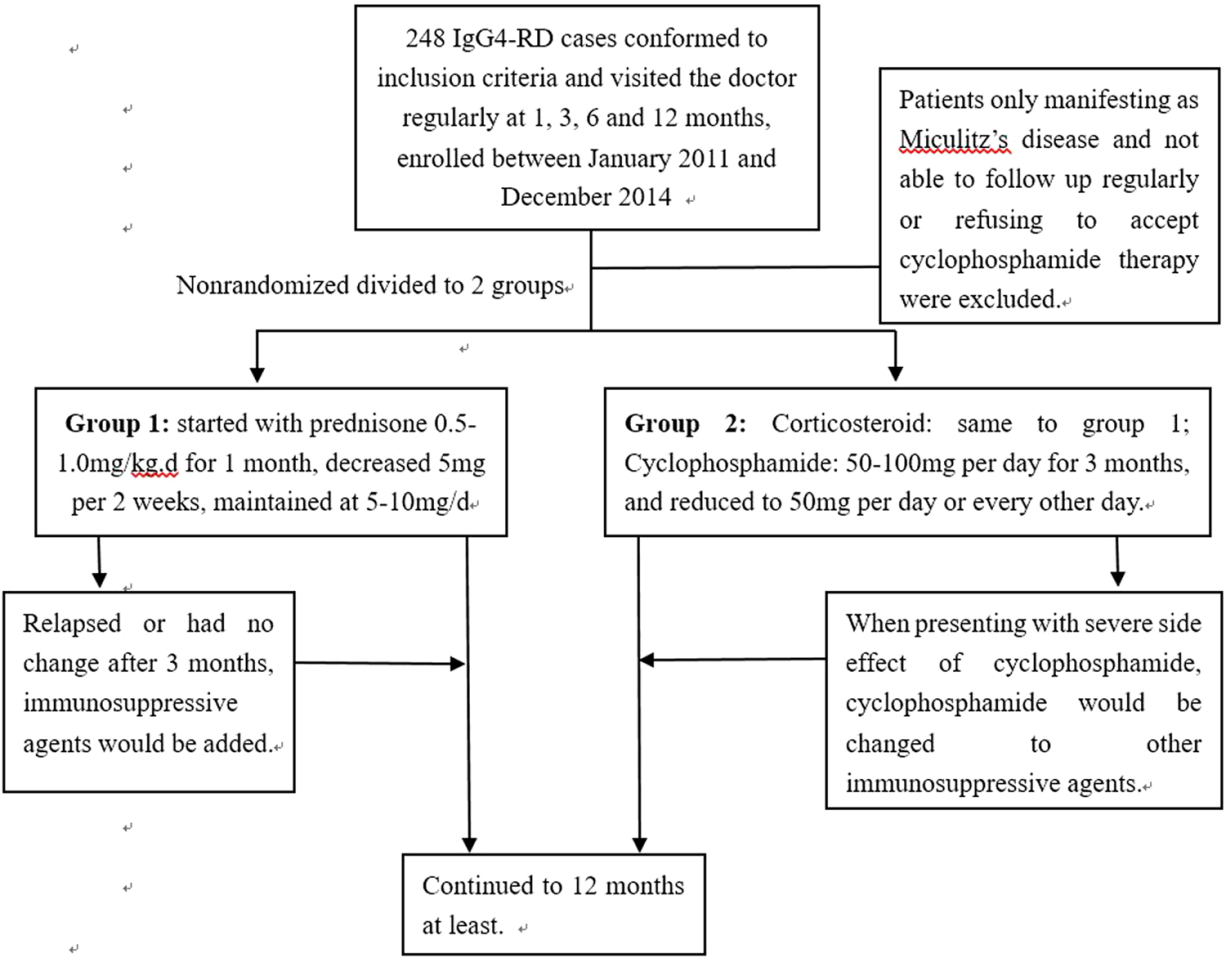
Management for IgG4-RD patients enrolled in this study.

Out of the 248 patients, 102 cases who conformed to inclusion criteria and visited the doctors regularly at 1, 3, 6 and 12 months were included in this study. Patients who didn’t attend this study including 27 cases fulfilling exclusion criteria, especially those without internal organs involvement (only manifested as Mikulicz’s disease) and 119 cases refusing to accept cyclophosphamide therapy or not able to follow up regularly. All patients enrolled in this study had been followed up for more than one year (from 12 months to 36 months) and nonrandomized divided into 2 groups, corticosteroid monotherapy and corticosteroid combined cyclophosphamide (CYC) treatment, shown in Fig. 1.

**Group I**: Corticosteroid: started with prednisone at 0.5–1.0 mg/kg.d for 1 month, decreased 5 mg per 2 weeks, maintained at 5–10 mg/d. No other immunosuppressant was used at initial therapy. If patients relapsed or had no change after 3 months treatment, one of the immunosuppressive agents would be added, including cyclophosphamide, azathioprine, methotrexate, leflunomide, mycophenolate mofetil or tripterygium wilfordii.

**Group II**: Corticosteroid: started with prednisone 0.5–1.0 mg/kg.d for 1 month, decreased 5 mg per 2 weeks, maintained at 5–10 mg/d; Cyclophosphamide: 50–100 mg per day for 3 months, and reduced to 50 mg per day or every other day. No other immunosuppressant was used at initial therapy. If patients presented with severe side effect of cyclophosphamide, cyclophosphamide would be changed to other immunosuppressive agents, such as azathioprine or mycophenolate mofetil.

### Inclusion criteria and exclusion criteria

Inclusion criteria: Aged 18 to 75 years old, all patients must meet the following diagnostic criteria of IgG4-RD (2011)^14^: (1) Swelling, sclerosing and inflammatory involvement of one or more organs, including sclerosing pancreatitis, sclerosing dacryoadenitis, sialadenitis (Mikulicz disease), sclerosing cholangitis, inflammatory pseudotumors, retroperitoneal or mediastinal fibrosis, interstitial nephritis, IgG4 related lung diseases, hypophysitis, hypertrophic pachymeningitis, inflammatory aortic aneurysm, lymphadenopathy, or other inflammatory conditions; (2) A high (>135 mg/dl) serum IgG4 concentration; (3) Specimens from patients who underwent biopsy showing characteristic histopathological findings of numerous infiltration of lymphocytes and plasma cell with obvious fibrosis, IgG4 + cells/IgG-positive cells >40% and >10 IgG4 + plasma cells/high-power field. Patients were classified into definite (1 + 2 + 3), probable (1 + 3) or possible (1 + 2) IgG4-RD.

Exclusion criteria: (1) Presenting only with Mikulicz’s disease without other internal organs involvements. (2) Serious infection; (3) Pregnancy or lactation; (4) Active viral hepatitis; (5) Serious cardiac insufficiency; (6) Meeting the criteria for rheumatoid arthritis, systemic lupus erythematosus, vasculitis or sarcoidosis; (7) Malignant disease; (8) Patients with fertility requirements.

### Assessment of Disease Activity and study endpoints

The IgG4-RD responder index (RI) was designed to assess disease activity per visit, using a scoring system from 0–4 for each organ system or site^15^. An IgG4-RD RI score ≥3 was recently used to identify patients with active disease.

Clinical response was classified into complete response, partial response, and no change at 1, 3, 6 and 12 months after treatment. Complete response was defined as an IgG4-RD RI <3 and declined ≥2 points after treatment. Partial response was defined as an IgG4-RD RI that declined ≥2 points but remained ≥3^10^. If IgG4-RD RI was 3 at initial and partial response was defined as 1 point decline after treatment. No change (NC) was defined as an absence of marked changes in mass sizes, organomegaly and/or symptoms and change of IgG4-RD RI <2 points.

Disease relapse was divided to clinical relapse or serological relapse; the former was defined as clinical symptoms recurred or imaging findings were worsened with or without IgG4 level increased, and the later was defined as serum IgG4 level elevated and IgG4-RD RI increased ≥1 after improvement with treatment, without clinical symptoms recurrence or imaging findings worsened.

Patients who achieved an IgG4-RD RI <3 or decline ≥2 points and successfully completed a glucocorticoid taper without relapse were considered in disease remission.

The primary end point was relapse rate at different periods during 12 months. The secondary end points included response rate, remission rate and adverse effects. Patients were evaluated at 1, 3, 6 and 12 months after the initial therapy of glucocorticoid with or without cyclophosphamide. Treatment efficacy was analyzed at different period and the patients could be changed to other therapies when he or she fulfilled the above-mentioned relapsing criteria.

### Laboratory tests, imaging studies and histological examination

Complete blood count (CBC), liver and renal function tests, erythrocyte sedimentation rate (ESR), C-reactive protein (CRP), serum immunoglobulin levels, IgG subclasses, total IgE levels, rheumatoid factor and antinuclear antibodies (ANAs) were tested. All patients underwent imaging examinations, including ultrasonography, thoracic and abdominal computed tomography (CT), or magnetic resonance imaging (MRI), or Positron Emission Tomography/Computed Tomography (PET-CT).

### Statistical Analysis

All parameters are described in the standard statistics, including mean, standard deviation etc. Comparisons between different groups were performed using the Chi-Square or t test. All statistical analyses were performed by SPSS version 19.0. A *P*-value < 0.05 was considered statistically significant.

## Results

### Characteristics and organ involvement of patients at baseline

Of the 102 patients diagnosed with IgG4-RD in this study, there were 80 males and 22 females, 52 cases had monotherapy of prednisone (Group I) and 50 patients received combination therapy with prednisone and CYC (Group II). Patient profiles of 2 groups at baseline are summarized in Table 1. There was no significant difference of clinical features at baseline between 2 groups, including the median age, the male to female ratio at diagnosis, organ involvement and laboratory characteristics etc. It was noted that the baseline IgG4-RD RI was almost same in 2 groups.

**Table 1:** Background of patients with immunoglobulin G4-related disease.

Character	Treated with Glucocorticoid (N = 52)	Treated with Glucocorticoid and CTX(N = 50)	P* < 0.05
Gender: Male: Female	40:12	40:10	0.811
Age (years) (median)	49.72 ± 14.01	53.52 ± 10.27	0.094
Fever	6(11.54%)	3(6.00%)	0.488
**Organ involvements**			
Number of involved organs(median)	4.71 ± 1.90	4.84 ± 2.00	0.741
1 to 3	14	12	0.912
4 to 6	29	28	
7 or more	9	10	
Lung	21(40.38%)	18(36.00%)	0.687
Bile duct	15(28.85%)	14(28.00%)	1.000
Retroperitoneum/abdominal aortitis	10(19.23%)	14(28.00%)	0.354
Prostate	8(14.81%)	14(28.00%)	0.149
**Serological features at baseline, median**			
Eosinophils	0.59 ± 0.21	0.67 ± 0.24	0.809
CRP (mg/l)	15.05 ± 4.67	14.03 ± 3.39	0.861
ESR (mm/h)	41,13 ± 4.67	38.65 ± 4.82	0.713
IgE(KU/L)	809.09 ± 184.67	918.27 ± 226.64	0.708
IgG4 (mg/dl)	13568.39 ± 1919.44	17462.52 ± 3019.99	0.263
IgG4-RD RI	14.52 ± 5.46	14.78 ± 5.64	0.813

Lymph node, salivary glands, pancreas and lacrimal glands were the most frequently affected anatomical sites in this study and there was no significant difference between 2 groups. In addition, retroperitoneum was also common involved sites in IgG4-RD, which were more frequent in Group 2 than that in Group I, but without statistical significance. There was no significant difference between 2 groups of less frequent disease localizations, including pachymeninges, nasal sinuses, kidneys and gallbladder (data not shown).

The mean ESR, CRP, serum IgG level, total IgE level and serum IgG4 level at baseline were all elevated in the 2 groups. Serum IgG4 level was higher in Group II than that in Group I, without significance (Table 1).

### Primary endpoint: relapse rate at 3, 6 and 12 month

In Group I, 52 patients were treated with glucocorticoids alone and in Group II, 50 patients were treated with combination of glucocorticoids and CYC. Almost all patients showed marked improvement of IgG4-RD RI and attained complete or partial response at 1 month in both groups.

**Figure 2:**
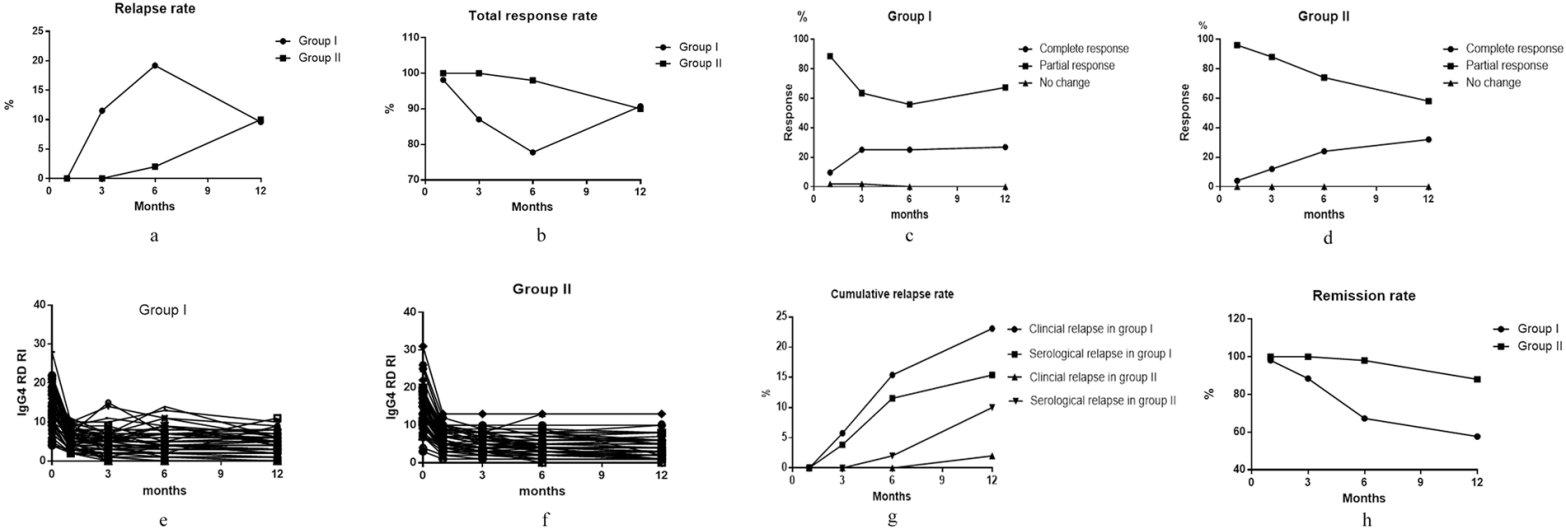
(**A**) Reslapse rate of 2 groups at different time point. (**B**) Total response rate of 2 groups at different time point. (**C**) Complete response, partial response and no change of the IgG4-RD patients in group I. (**D**): Complete response, partial response and no change of the IgG4-RD patients in group II. (**E**) Analysis of IgG4-RD RI before and after treatment in Group I. (**F**) Analysis of IgG4-RD RI before and after treatment in Group II.(**G**). The cumulative relapse rate of Group I and Group II. (**H**) Remission rate of 2 groups at different time. Figure 2: IgG4-RD patients, including Group I (with corticosteroid monotherapy) and Group II (with combination therapy of corticosteroid and cyclophosphamide) were treated and followed up for at least 12 months; the percentages of relapse rate and the total response rate were showed respectively in Fig. 2A and B. The percentages of complete response, partial response and no change rates in Group I and Group II were illustrated in Fig. 2C and D. The changes of IgG4-RD RI before and after treatment in Group I and Group II were showed in Fig. 2E and F. Figure 2G showed the cumulative clinical and serological relapse rate of Group I and Group II. Figure 2H showed the remission rate of 2 groups at different follow-up time.

However, the relapse occurred with steroid tapering. During one year follow up period, the accumulative relapse rate was significantly higher in Group I than in Group II (Fig. 2A). In Group I, accumulative 20 patients suffered relapse, accounting for 38.5%, including 12 (23.1%) cases of clinical relapse and 8 (15.4%) cases only with serological relapse. Whereas, disease relapse only occurred in 6 patients from Group II, accounting for 12.0%, in which only 1 patient had clinical relapse and 5 patients had serological relapse.

As for the time to relapse, in Group I, 5 patients relapsed at 3 months; 9 patients recurred at month 6; 6 patients had recurrence at 12 months and all above got good response after added immunosuppressive agents, shown in Fig. 2A–F. However, in Group II, only 1 patient relapsed at month 6 and other 5 patients got recurrence at 12 months. The relapsing rate at month 3, 6 and 12 were 9.6%, 17.3%, 11.5% in Group I and 0%, 2.0%, 10.0% in Group II, shown in Fig. 2G. There was a statistically significant difference of the mean time to flare between 2 groups, which were 7.05 ± 3.55 (3~12) months in Group I and 11.00 ± 2.45 (6~12) months in Group II respectively, P = 0.018, shown in Fig. 2A.

### Response rates at 1, 3, 6 and 12 month

The total, complete and partial response rate were shown in Fig. 2B,C and D. Glucocorticoid monotherapy had almost the same efficacy as combination therapy at initial treatment, while there was significant difference of therapy effect afterwards. Disease remission was obtained in 31/52 (59.6%) patients in group I and 44/50 (88.0%) in group II during 1 year, shown in Fig. 2H.

In Group I, thirty-one subjects (13 cases achieving CR and 18 cases achieving PR) had good response and could complete glucocorticoids tapering without experiencing flares; 20 relapsing patients accepted additional treatments with immunosuppressive agents after relapse and got good response once again, including azathioprine, methotrexate, mycophenolate mofetil etc. There was no difference of the total response rate between 2 groups at 12 months, shown in Fig. 2B, indicating that immunosuppressive agents had good effect on relapsing patients treated with glucocorticoid monotherapy. One patient had no change at 3 months therapy treated with prednisone monotherapy, who achieved partial response at 1 year after added with azathioprine.

In the 44 patients with no flare in Group II, 16 patients achieved CR and 28 patients attained PR at 12 months, accounting for 32.0% and 56.0% respectively, which was increased as time of therapy going on from 3 months to 12 months, shown in Fig. 2D and F. Of the 6 relapsing cases treated with glucocorticoids and CYC, 1 patient had organ recurrence, presenting with pancreatitis swelling who had methylprednisolone pulse therapy and got disease improvement once again; the other 5 patients only had serological relapse, who didn’t change treatment and kept in a stable condition for a long time.

Based on the evidence provided so far, almost all IgG4-RD patients were sensitive to glucocorticoids therapy, but clinical or serological relapse might occur in a portion of patients within one year period who received prednisone monotherapy. Prednisone combined with CYC decreased the relapse rate significantly. All of relapsing patients with prednisone monotherapy were sensitive to immunosuppressive agents.

### Analysis of differences in clinical and serological characteristics between ‘relapsing’ and ‘non-relapsing’ patients treated with glucocorticoids monotherapy

The organ involvement was evaluated by symptoms, signs, radiographic or other imaging examinations or tissue biopsies. Compared to non-relapsing patients, relapsed patients had higher IgG4 levels, more organ involvements, and higher IgG4-RD RI at baseline (p = 0.023, P = 0.009 and P = 0.002, respectively), whereas there was no difference in baseline levels of other epidemiological and serological features, including ESR, CRP, and IgE, shown in Table 2. Most of patients (7/8, 87.5%) got recurrence when he or she had more than 6 organs involvement at the onset of therapy.

**Table 2:** Differences in clinical and serological features at baseline between ‘relapsing’ and ‘non-relapsing’ patients in Group 1.

Character	Relapsing patients (N = 20)	Non-relapsing patients (N = 31)	P* < 0.05
Gender: Male: Female	15:5	24:7	1.000
Age (years) (median)	48.57 ± 13.12	49.20 ± 14.78	0.879
Fever	4(20.00%)	2(6.45%)	0.195
**Serological features at baseline, median**			
Eosinophils	0.52 ± 0.17	0.78 ± 0.39	0.627
CRP (mg/l)	13.89 ± 11.15	13.28 ± 4.94	0.953
ESR (mm/h)	37.18 ± 8.42	42.96 ± 6.59	0.589
IgE	766.34 ± 178.09	849.18 ± 322.76	0.827
IgG4 (mg/l)	19908.86 ± 3428.35	10348.89 ± 2392.37	0.023*
Number of involved organs	5.50 ± 2.06	4.13 ± 1.57	0.009*
1 to 3	4	10	0.010*
4 to 6	9	20	
7 or more	7	1	
IgG4-RD RI	17.14 ± 4.87	12.60 ± 5.12	0.002*

Of the 20 relapsing patients treated with glucocorticoids monotherapy, bile duct, lacrimal glands and lymph node were commonly relapsed organs, shown in Table 3. In addition, recurrence also happened at paranasal sinus, skin, pancreas, retroperitoneum and salivary glands during the therapy period. Only 1 patient had organ recurrence in Group II, manifesting as pancreas swelling. Therefore, it was concluded that combined treatment of CYC remarkably reduced organs recurrence.

**Table 3:** Organs recurrence in relapsing patients with IgG4-RD.

Involved organs	Number of cases in relapsing patients	
	Group 1	Group 2
	Organs recurrence/Involved organs	Organs recurrence/Involved organs
Salivary glands	3/32(8.82%)	0/25 (0.00%)
Lacrimal glands	3/24(12.50%)	0/19 (0.00%)
Lymph node	4/32(11.76%)	0/26 (0.00%)
Pancreas	2/24(8.33%)	1/22 (4.55%)
Retroperitoneum/abdominal aortitis	1/10 (10.00%)	0/14 (0.00%)
Paranasal sinus	2/17 (11.76%)	0/9(0.00%)
Bile duct	2/15 (13.33%)	0/14(0.00%)
Lung	0/21 (0.00%)	0/18 (0.00%)
Skin	1/8 (12.50%)	0/6(0.00%)
Prostate	0/8 (0.00%)	0/14(0.00%)

### Follow-up and outcomes

In this study, patients were monitored for a median follow-up period of 24.45 ± 7.37 (15~36) months. After 1 year, CYC could be changed to other immunosuppressant agents, including methotrexate, leflunomide, azathioprine, mycophenolate mofetil. Follow-up time or interval did not differ between IgG4-RD patients treated with glucocorticoids alone and combination therapy of CYC.

All of the 20 relapsing IgG4-RD patients in Group I, attained CR or PR added with immunosuppressant agents afterwards, shown in Table 4. One relapsing IgG4-RD patient developed to diffuse large B cell lymphoma after 30 months follow-up.

**Table 4:** Follow-up and outcomes of relapsed IgG4-RD patients in Group 1.

Patient No.	Organ recurrence or/and IgG4 elevation	Added immunosuppressant agents	Relapse time of prednisone monotherapy	Outcome	Follow-up
1	Salivary glands + lymph nodes	Methotrexate	12	CR	36
2	Pancreas + bile duct	Cyclophosphamide	6	CR	24
3	Retroperitoneum	Cyclophosphamide	3	PR	24
4	IgG4 elevation	Cyclophosphamide	6	CR	30
5	Lacrimal glands + lymph nodes	Leflunomide	6	Lymphoma	30
6	Lacrimal + salivary glands	Cyclophosphamide	6	PR	36
7	IgG4 elevation	Cyclophosphamide	6	PR	24
8	IgG4 elevation	Azathioprine	12	CR	24
9	Lacrimal glands	Methotrexate	3	PR	36
10	IgG4 elevation	Cyclophosphamide	12	CR	24
11	IgG4 elevation	Cyclophosphamide	6	PR	24
12	IgG4 elevation	Azathioprine	3	PR	18
13	Salivary glands + paranasal sinus + skin	Azathioprine	12	PR	15
14	IgG4 elevation	Mycophenolate Mofetil	6	CR	24
15	Lymph nodes	Methotrexate	6	PR	18
16	Lymph nodes	Cyclophosphamide	3	CR	18
17	Pancreas	Cyclophosphamide	6	PR	15
18	IgG4 elevation	Tripterygium wilfordii	12	CR	36
19	Bile duct	Azathioprine	3	PR	15
20	Paranasal sinus and IgG4 elevation	Cyclophosphamide	12	PR	18

### Adverse reactions

Two patients switched from CYC to mycophenlate mofetil because of liver toxicity in Group 2. GC-induced diabetes mellitus was newly diagnosed in 8 patients, 1 of whom required insulin therapy. No case showed severe infection and no patient discontinued treatment due to infection, shown in Table 5.

**Table 5:** Adverse events observed during treatment in patients with IgG4-RD.

Adverse events	Number of cases (%)	
	Group 1	Group 2
Glucose intolerance	8	7
Newly diagnosed with DM	5	3
Aggravation of DM	3	4
**Infection**		
Upper respiratory tract infection	1	2
herpes zoster	1	1
Pneumonia	0	0
Tuberculosis	0	0
Liver dysfunction	0	2
Leukopenia	0	0
Gastrointestinal reaction	0	2
Hemorrhagic cystitis	0	0

## Discussion

Although glucocorticoids therapy has been shown effective for patients with IgG4-RD, few prospective studies have analyzed the effect of CYC or other immunosuppressant agents in this disease. In the first international consensus guidance statement on the management and treatment of IgG4-RD, the opinion among experts on the statement of using of steroid-sparing agents was split (46% agreement)^16^, because till now there has been no good evidence for immunosuppressant agents in treating IgG4-RD. This study was a prospective non-randomized controlled clinical trial, in which we aimed to investigate the therapeutic effect of glucocorticoids and CYC versus glucocorticoids monotherapy.

The major salivary glands, lymph nodes and pancreas were the most frequently affected anatomical sites in this study, similar to previous reports^10, 17^. Clinical manifestations were protean and varied according to the different organ involvement. There was no significant difference of clinical features at baseline between patients treated with glucocorticoids monotherapy and prednisone combined with CYC. Not only did disease flares occur in 39% of the patients over one year of treatment despite receiving ongoing prednisone of not less than 5 mg/day, it also seems that only 25% (13 of 52 patients) achieved complete remission in Group I, which suggested the poor ability of glucocorticoids monotherapy to achieve sustained disease control of IgG4-RD. However, the patients treated with glucocorticoids and CYC had significantly higher incidence of CR rate, lower occurrence of relapse rate and longer relapse time than glucocorticoids monotherapy. Therefore, glucocorticoids combined CYC treatment were more efficacious and preventing from disease flare better than glucocorticoids monotherapy.

Bile duct, lacrimal glands, lymph node and pancreas were frequently relapsed organs in Group 1, accounting for 13.33%, 12.50%, 11.76%, and 8.33% respectively, shown in Table 3, while only 1 patient had pancreas recurrence in Group 2. No certain organ involved predicted disease relapse to treatment, but most of patients relapsed after 3 months when having more than 6 organs involved who were treated with prednisone alone, and relapsing patients had significantly higher IgG4 levels than non-relapsing patients at baseline. As a consequence, it was clear that combined treatment of CYC decreased organs recurrence greatly and maintained disease remission. The patients with more than 6 organs involved should accept treatment of glucocorticoids and immunosuppressant agents at the beginning to prevent relapse. Besides, serum IgG4 level should be considered as a useful tool for therapy purpose.

All the relapsing IgG4-RD patients in Group 1 accepted immunosuppressant agents therapy after disease flare, achieving CR or PR for a long time; only 1 relapsing IgG4-RD patient developed to diffuse large B cell lymphoma. No difference of the total response rate was found between 2 groups at 12 months. Hence, we concluded that immunosuppressant agents had a good effect on most of relapsing IgG4-RD patients accepting therapy of glucocorticoids alone at initial.

Long term cyclophosphomide toxicity was observed in our patients. Patients were monitored for a median follow-up period of 24.45 ± 7.37 (15~36) months. After 1 year, CYC could be changed to other immunosuppressant agents, including methotrexate, leflunomide, azathioprine, mycophenolate mofetil etc. No long term toxicity such as bladder cancer was reported.

Our study also has some limitations, due to its non-randomized trait, leaving room for potential bias, but it worked almost as well as a matched study because the baseline characteristics showed no statistical differences between the 2 groups. The other concerning aspect is that the study is not blinded to doctors, which might affect disease activity and relapse assessment.

In conclusion, glucocorticoids monotherapy is an efficient treatment for IgG4-RD patients, having almost the same effect as glucocorticoids and CYC at the initial stage, but glucocorticoids combined CYC treatment had better effect and lower relapse rate than glucocorticoids monotherapy overtime. The patients with more than 6 organs involved should receive glucocorticoids and immunosuppressant agents treatment at the onset. Immunosuppressant agents were efficacious for most relapsing patients treated with glucocorticoids alone.
